# Evaluation of Methods for *De Novo* Genome Assembly from High-Throughput Sequencing Reads Reveals Dependencies That Affect the Quality of the Results

**DOI:** 10.1371/journal.pone.0024182

**Published:** 2011-09-07

**Authors:** Niina Haiminen, David N. Kuhn, Laxmi Parida, Isidore Rigoutsos

**Affiliations:** 1 Computational Biology Center, IBM Thomas J. Watson Research Center, Yorktown Heights, New York, United States of America; 2 Subtropical Horticulture Research Station, Agricultural Research Service (ARS), United States Department of Agriculture (USDA), Miami, Florida, United Sates of America; University of Chicago, United States of America

## Abstract

Recent developments in high-throughput sequencing technology have made low-cost sequencing an attractive approach for many genome analysis tasks. Increasing read lengths, improving quality and the production of increasingly larger numbers of usable sequences per instrument-run continue to make whole-genome assembly an appealing target application. In this paper we evaluate the feasibility of *de novo* genome assembly from short reads (≤100 nucleotides) through a detailed study involving genomic sequences of various lengths and origin, in conjunction with several of the currently popular assembly programs. Our extensive analysis demonstrates that, in addition to sequencing coverage, attributes such as the architecture of the target genome, the identity of the used assembly program, the average read length and the observed sequencing error rates are powerful variables that affect the best achievable assembly of the target sequence in terms of size and correctness.

## Introduction

Recent advances in massively parallel genome sequencing provide a cost-effective potential alternative to the traditional Sanger method [1]. However, the realized increased throughput and lower cost comes at the expense of read length and accuracy. Indeed, the currently reported average read lengths [2], [3], [4] and are between 75 and 100 nucleotides (nts) for Illumina/Solexa GAII, 50–75 nts for Life/APG SOLiD and 330 nts for Roche/454 GS FLX Titanium, versus up to ∼1,000 nts for Sanger sequences. Mate paired protocols generate read lengths up to 75 nts for Illumina/Solexa and 60 nts for SOLiD. For individual reads, the estimated error rates, as a fraction of the generated bases, are approximately 1% (Solexa and 454 Titanium) versus up to 1% in Sanger sequencing [5], [6].

The *de novo* assembly of high-throughput sequencing reads into high quality reference sequences will increase our knowledge of important organisms and yield important advantages in many genome analysis tasks. The number of *de novo* short read genome assembly tools has been increasing steadily. A (partial) list of the tools that are currently available can be found at http://seqanswers.com/wiki/Software/list. Many recent methods opt to represent reads as *k*-mers, i.e. words of length *k*. A graph is constructed from all *k*-mers occurring in the input reads, and, finally, the reads are threaded into paths through the graph: these paths represent alternative, compatible assemblies of the input sequences. The graph representation allows for a compact representation and processing of the input whereas its size depends on the genome size and the number of *k*-mers. Representative methods in this category include Euler-SR [7], Velvet [8] and Allpaths-LG [9]. Other schemes are based on a more traditional overlap and contig extension approach and include the Edena method [10], Sharcgs [11] and Vcake [12]. These assemblers have been designed to handle small genomes, such as bacteria, and may not be directly applicable on larger more complex genomes. The ABySS assembler features a distributed de Bruijn graph, employing parallel computing to assemble larger genomes [13]. Recently, SOAPdenovo, a variation of SOAP [14], was applied on the human genome using single-end and paired-end reads of 35–75 nts, and achieved 87% genome coverage [15]; the achieved contig N50 size was 7.4 kb at best, thus the assembly is highly fragmented. Reviews of high-throughput sequencing technologies and assembly tools can be found elsewhere [16]. In addition to short read assemblers, there are specialized tools for assembling longer pyrosequencing reads (i.e., from the 454 technology), such as CABOG [17].

Even the current assemblies of important model organisms are subject to continuing finishing processes; for example, recent improvements in the mouse genome assembly added 267 Mb of previously missing or misassembled sequence [18]. Efforts to finish shotgun-based vertebrate genome assemblies are further complicated by a high amount of species-specific variability regarding mis-assembly and gap characteristics, making it challenging to apply standardized finishing strategies [19]. Some promising approaches for tackling the problem of high-throughput sequence assembly by using a closely related reference genome have been proposed, including gene-boosted assembly [20] and assisted assembly [21].

Further complicating the picture are the error profiles of the various new sequencing technologies and associated platforms. These profiles have not been adequately characterized in the literature, and they appear to be changing with every iteration of a given platform. To the best of our knowledge, there has been only anecdotal evidence on the impact of the resulting error rates on the available assembly tools.

The work described below has been motivated by our participation in the USDA/MARS/IBM consortium whose goal is to sequence and analyze the genome of *Theobroma cacao* (*T. cacao*) with an estimated length of approximately 400 M bases. One of the questions that arose in the context of the project is whether the capabilities of today's high-throughput sequencing platforms are such that a *de novo* assembly of *T. cacao* from short reads is feasible.

Short read lengths present formidable challenges for de novo genome assembly because several valid alignments can exist for a given set of very short sequences. In principle, one of those possibilities corresponds to the target genome sequence.

The number of alignment possibilities depends on the length of overlap that is required to align the ends of two sequences. There are also limits to the quality of the assembly results that can be achieved: it is not possible to determine the exact size of tandem repeats that are longer than the read length (e.g. ATCATC…ATC). Also distinguishing between two near-exact copies of the same repeat in different parts of the genome may not be possible, since short reads do not necessarily provide enough sequence context to determine the relative position of the read in the genome.

Adding information from paired reads with large insert sizes can potentially assist in determining the correct origin of repeat copies and can also help in scaffolding contigs into longer stretches of ordered sequence (with gaps of unknown sequence and potentially unknown length still remaining).

Highly fragmented assemblies with repeat expansions and collapses, and falsely joined sequences can be characteristic of short read assembly results on repeat-rich genomes. Clearly, these complications continue to persist even in the presence of high sequencing coverage.

As outlined above, there are several challenges and sources of error associated with genome assembly from short sequencing reads. Nonetheless, high-throughput short reads have proven useful in several assembly tasks. Short reads have been combined with other sources of data to generate and improve *de novo* genome assemblies; examples include the rice pathogen *Pseudomonas syringae* [22], the forest pathogen *Grosmannia clavigera* [23], plant chloroplast genomes [24], and also *Arabidopsis thaliana* strains [25]. Recently, individual human genome datasets were assembled into fragments by ABySS [13] and SOAPdenovo [15] yielding numerous small contigs covering in total up to 80% of the human genome. The first example of researchers having employed high throughput sequencing alone to assemble a large animal or plant genome was recently reported for the giant panda genome [21]. However, it should be pointed out that the ‘true’ quality of the resulting assembly remains unclear, as it was estimated by employing comparisons to the dog genome, a limited amount of pre-existing mRNA annotations, and various repeat estimation techniques.

A fundamental concern when performing *de novo* genome assembly stems from limited confidence in the assembled contigs since they represent only one possible way of mapping the sequence fragments to contiguous sequences. There have been efforts to computationally simulate certain aspects of the assembly process in order to gauge the performance of existing approaches. For example, benchmarking datasets and assembly evaluation for metagenomics sequencing data have been presented [26]. Also, the original publications that describe a novel assembly algorithm typically include some validation and comparison with some of the existing methods [8], [10], [13]. Some very recent studies compare short read assembly methods under various conditions and for various types of genomic input [27], [28]. Obviously, having a few long contigs is desirable; however, an equally important consideration is the *correctness* of the contigs.

In this paper, we study *de novo* assembly through simulation. From several reference sequences, ranging from viral to plant, we generated simulated reads with lengths between 50 and 100 nts, these lengths being typical of the current short-read generating platforms. We introduce and employ a protocol for evaluating a *de novo* assembly strategy for a genome for which a reference sequence does not exist. Our protocol calls for generating simulated sequencing reads from a carefully chosen related reference genome, assembling them *de novo* and finally aligning the assembled contigs to the reference and quantifying the erroneously and correctly assembled nucleotides. From the results, we can determine whether a sequencing and assembly strategy employed in the simulation would yield meaningful results on the related unsequenced genome. By injecting errors at varying rates into the reads, and by investigating different degrees of sequencing coverage, we obtain limits to the error that the assembler tolerates, and determine which coverage ranges are most useful. Finally, we examine the extent of improvement that results from the use of paired read information.

It is important to point out that the employed simulation framework represents ideal conditions for assembly. In real data, among other complicating factors such as non-uniformity in the lengths of obtained reads, true sequencing coverage varies across the template and some regions may even fail to be captured in the sequencing process. Thus assembling real data poses additional difficulties; in that regard, our simulations represent an upper limit (*best* case scenario) of what can be achieved with a given average sequencing coverage.

## Results

### Assembly programs

We studied the performance of six popular genome assembly tools that have been designed to handle short sequencing reads (∼50 nt). For our analysis, we compared tools that we were able to port to and run in our computing environment: *ABySS* [13], *Edena* [10], *Euler-USR* [7], *Sharcgs* [11], *Vcake* [12], and *Velvet* [8]. In order to have control over the simulation and to simplify comparisons, we assumed that each base of the input sequences is of sufficient quality for assembly.

### Benchmark datasets

The simulated benchmarks were generated as follows:

#### Benchmark I: Effect of sequence size and error on assembly

A set of reads 50 nts long was generated from each genomic reference sequence, and errors were introduced uniformly and at random into *E*% of the read positions. Here *E* assumed the values 0%, 1%, and 5%. As an example, “applying” a 1% error rate on 500,000 reads of length 50 nts will affect 500,000*50*0.01 = 250,000 out of a grand total of 25,000,000 bases. The reads comprising a given input were chosen randomly and uniformly from the reference genome. Thus, each sequence position is expected to have the same coverage, which is the ideal case rather than what would be encountered in practice. In real sequencing experiments the coverage would be non-uniform, which makes the assembly more challenging, and also the errors would be concentrated towards the ends of the reads. There would also exist insertion and deletion errors, which would further complicate assembly. Furthermore, heterozygosity in the genomes of real sequenced organisms would generate variability in the sequencing reads and make genome assembly more challenging. As we are looking into the limits of short-read assembly in near-ideal conditions, we did not include many of the realistic complications one encounters when dealing with real sequencing data.

The synthetic reads, 50 nts long, represent 50× uniform coverage of the source genome. An exception is the dataset having 188× coverage of 30 nts long reads from a *D. melanogaster* BAC which we did not generate but included since Sharcgs performance had already been evaluated on this dataset. Assemblies of error-free inputs correspond to an ideal scenario, an error rate of 1% approximates real data and an error rate of 5% represents an extreme of the simulation.

For the *O. sativa* 4 Mb dataset, we evaluated the impact of small changes in error rate on the quality of the assembly by generating additional sets of reads with error rates *E* equal to 0.5%, 1.5%, 2% and 2.5%.

#### Benchmark II: Effect of read length and error on assembly

This benchmark focused on evaluating the impact that increased read length can have on the quality of the assembly. We generated 50× coverage of reads with lengths 75 nts and 100 nts from the *O. sativa* reference sequence that had proved challenging due to its regions of repeat sequences.

#### Benchmark III: Effect of coverage and error on assembly

This benchmark focused on evaluating the impact that increased coverage can have on the quality of the assembly. We generated additional sets of reads corresponding to 10×, 20× and 100× coverage of the *O. sativa* segment.

#### Benchmark IV: Effect of paired end data and error on assembly

Using the *O. sativa* segment, the sets comprised 50× coverage of paired reads of 50 nts originating from ends of fragments of sizes 500, 1000, 3000 and 5000 nts with variation ±50 nts. We assumed perfect pairing and experimented with several coverage combinations of unpaired and paired reads. We also replicated some Velvet assembly results several times to explore the robustness of the results across runs on independently generated data.

### Assembly evaluation

**Figure 1:**
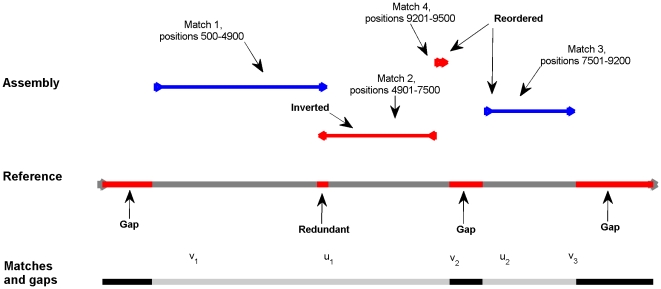
Illustration of assembly errors. A single contig is aligned against the reference sequence, observed assembly mistakes are shown in red. The contig has 4 matches against the reference. Match 1 is the longest one, and it defines the match window coordinates and orientation. The first 157 positions in the match window do not have contig matches, corresponding to an insertion. Match 1 and Match 2 have overlap, corresponding to redundant positions in the reference. Match 2 is inverted, it has opposite orientation compared to the match window. The latter half of Match 3 is outside the match window, corresponding to a relocation. Match 4 and Match 3 have incorrect order, relative to their contig positions, this corresponds to a reordering. Gaps and redundancies are also shown on the reference sequence.

We use two types of features to evaluate assemblies: correctness scores and size statistics. See Methods for details, and Fig. 1 for an illustration of different types of assembly errors.

1. Correctness scores. We consider five scores that reflect fundamental aspects of assembly correctness: inversion, insertion, redundancy, relocation, and reordering. For each score, value 1 is best and 0 worst.
2. Size statistics. We consider largest contig length, mean contig length, contigs sum, coverage, N50 contig size, and a match score. Higher values of size statistics are more preferable than lower values.

We next present and briefly discuss the results we obtained in our four benchmarks for each of the six types of genomic input where possible. The runs with assemblers Euler-USR, Sharcgs, and Vcake did not complete without errors or within a reasonable time (several hours) for the *O. sativa*, *E. coli* and *S. cerevisiae* datasets and are not present in the evaluation.

### Benchmark I Results: Effect of genome and error on assembly

**Figure 2:**
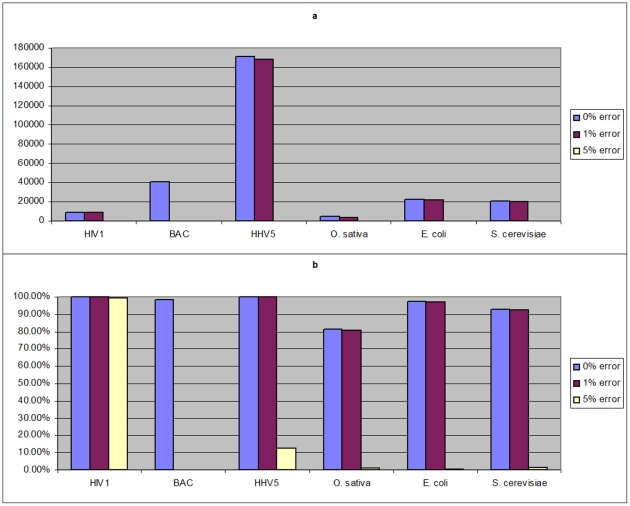
N50 contig size and genome coverage. The best values among all the studied assembler on a given reference sequence and error rate are reported. a) N50 contig size is shown for all studied sequences, with different error rates for the 50 nt reads at 50× coverage. Sequences are ordered from smallest (HIV1) to largest (*S. cerevisiae*). BAC data has 30 nt reads with 0.6% error, its results shown under 0% error label. When N50 size is zero it indicates the sum of contig lengths was less than 50% of reference sequence length. b) Percentage of reference genome that is covered by the assembly is shown for all studied sequences.

The results are summarized in Fig. 2 showing N50 contig size (a) and percentage of reference covered (b), for the best values achieved among the assemblers. The *O. sativa* sequence shows smallest N50 contig size and genome coverage, though the *E. coli* and *S. cerevisiae* genomes have larger sizes. About 90% of *E. coli* and *S. cerevisiae* genomes are covered by assemblies (when 0–1% sequencing errors are present), whereas coverage of the *O. sativa* sequence is ∼80%. The numeric data are presented in Tables S1, S2, S3, S4, S5, S6, where it is also clear that the correctness scores (reordering, inversion, redundancy, relocation, insertion) for all genomes are mostly near 1, except for the HIV1 genome with its major assembly mistakes.

It is evident that besides the case of the small HIV1 genome assembly, the rice sequence is the most challenging to assemble. Given this observation and our interest in plant genomes in conjunction with the *T. cacao* sequencing project, we used the rice sequence in the remaining Benchmarks II–IV

#### HIV1

**Figure 3:**
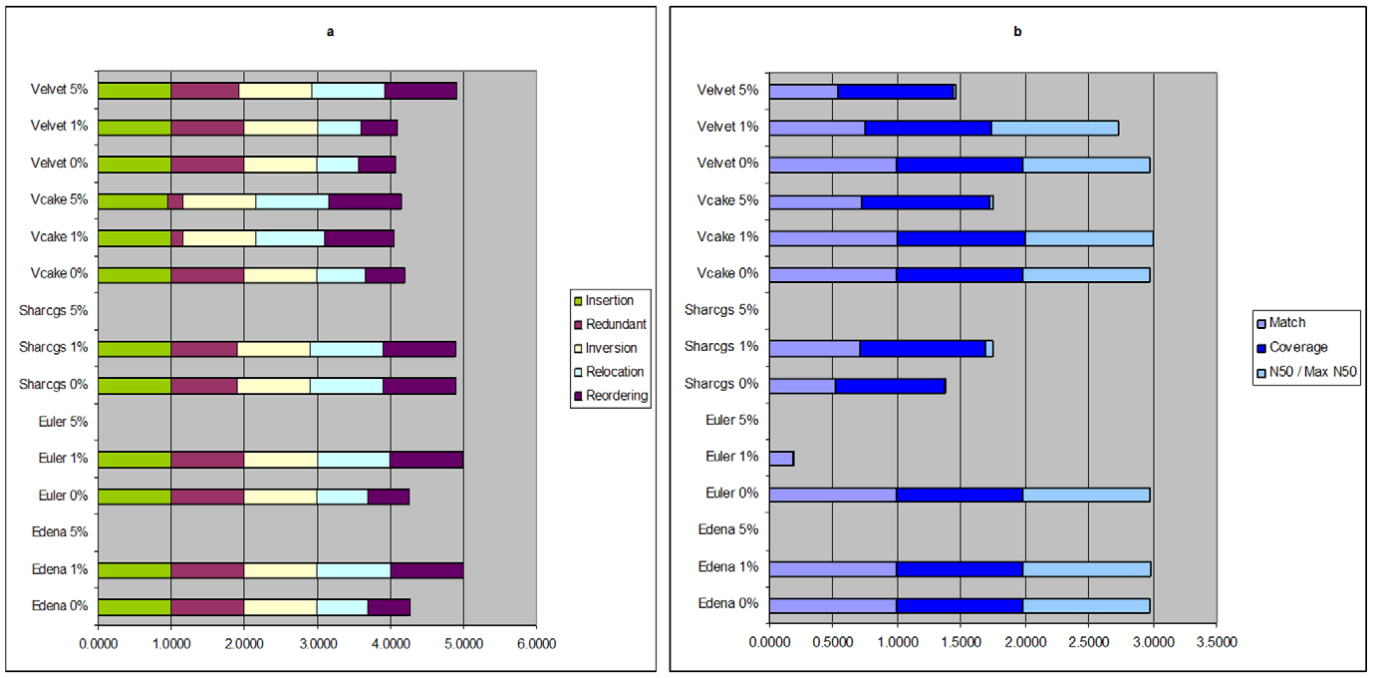
Correctness and size statistics for HIV1 assemblies. Assembly statistics are shown for all assemblers and various read error rates for HIV1 assemblies. a) Correctness scores, b) Coverage, and N50 divided by genome size.

Since the genome is only 9,181 bp, we consider all contigs at least 100 nt long (for all other genomes we use 1,000 nt as minimum contig length to avoid evaluating uninformative short fragments). In the case of error-free reads, all tools generated exactly one contig with 100% coverage, except ABySS (no contigs) and Sharcgs (52 contigs); see Table S1. The length of the assembled contig was close to the correct genome length, and it almost completely covered the genome, but with with poor relocation and reordering scores, indicating chimeric assembly mistakes. This is pretty notable considering the small genome length, the level of coverage and the fact that the reads were error-free. Presumably, the obtained poor performance results from the internal structure of the virus, which includes ∼600 bp long terminal repeat (LTR) elements at 3′ and 5′ ends of the genome. Interestingly, when 1% error is introduced, the repeats become a more manageable challenge for Edena and its results improve. At a 5% error in the input, only Velvet generates reasonable results. ABySS does not output any contigs at all: the repeats cause the genome to appear circular and therefore it is unable to produce any results. Fig. 3 shows (a) correctness and (b) size statistics for the assemblies. According to the correctness scores, Edena with 1% read errors provides the best assembly, and its size statistics are also near perfect. However, with 5% read errors Velvet's results are mostly correct but exhibit poor size statistics. On the other hand, for Vcake and reads with 1% errors the picture is the opposite. This demonstrates that correctness and size do not always go hand in hand.

#### *D. melanogaster* BAC

This BAC was described with the *Sharcgs* assembler (Dohm et al. 2007) and it has 0.6% errors introduced in the 30 nt simulated reads. This input is the type of example sequence on which many short read assembly tools have been tested and can be characterized as an “easy” case: indeed, it is a relatively short sequence, contains no complex internal structure, and there are only a few errors in the reads. There were 500,000 reads of length 30 nts corresponding to 188× coverage of the 80 kb sequence. Each tool assembled the sequence into 5–34 contigs, with the size of the largest contig ranging from 4,365 nts (Euler) to 40,884 nts (Sharcgs); see Table S2. Assemblies covered at least 98% of the source genomic sequence, with the exception of Euler (77%). Sharcgs had the highest N50 and match score, indicating the least fragmented assembly. In summary, and except for Euler, all tools performed well on this input.

#### HHV5

The genome of the human herpesvirus 5 has a size of 230 kb. Each tool assembled the error-free reads into 3–6 contigs; the size of the largest contig was around 170 kb. The genome coverage by the assemblies was close to 94%. At an error rate of 1% ABySS, Edena and Euler produce similar good results, while the other assemblers' performance deteriorates, as seen in the largest contig and N50 contig sizes. At 5% almost none of the reference is covered; see Table S3.

#### *O. sativa*

We focused on a 4 Mb region from *Oryza sativa* (rice) chromosome 12, which corresponds approximately to a BAC pool size suggested in a recent rice *de novo* assembly strategy [29]. In our computing environment, only ABySS, Edena, and Velvet were capable of completing the assembly, on *O.sativa* or any of the source genomic sequences described below. Assembly correctness scores were near perfect. N50 and match scores were very similar for all assemblers, with Velvet performing slightly better than the rest when read error rate was increased. For error-free reads, assemblers produced around 840 contigs – see Table S4 – with the longest contigs being 30 k nts for all tools. The assemblies covered at most 81% of the reference sequence, leaving more than 800 kb of the reference uncovered. Assemblies of reads at 0.5–2% error rates were similar to the error-free case; however, working with reads at a 5% error led to almost no results. The results of ABySS, Edena and Velvet were very similar, though none of them succeeded in assembling more than ∼80% of the reference sequence even without any sequencing errors present.

#### *E. coli*

The assemblers ABySS, Edena and Velvet generated around 330 contigs with the size of the largest contig being about 128 k nts; see Table S5. Velvet's assembly covered most (96.9%) of the reference sequence, leaving *uncovered* 110 k nt. The results obtained at an error rate of 1% show reduced N50 and max contig size for Edena and Velvet. On the other hand, at a 5% error rate, less than 1% of the genome is covered.

#### *S. cerevisiae*

Baker's yeast with a genome size of 12 Mb represented our final test set. In this case, we only report results by Velvet and Abyss as the other tools failed to finish the assembly. The assemblies are consistent up to 1% injected error, resulting in source input coverage of 92%, and up to 1,147 contigs; see Table S6.

### Benchmark II Results: Effect of read length and error on assembly

Assembly statistics with read lengths {50, 75, 100} nts for the *O. sativa* 4 Mb segment are shown in Table S7. The results are nearly identical across read lengths with 0–1% errors in the reads, except for Velvet with 1% read errors, were increasing read length actually decreases coverage. This suggests that the evaluated algorithms and parameters are best suited for 50 nts reads. It may well be the case that the k-mer length and other algorithmic parameters require tuning to obtain best results as the average read length changes.

### Benchmark III Results: Effect of sequencing coverage and error on assembly

The effect of varying the sequencing coverage from 10× to 100× is shown in Table S8. With read error rates 0–1%, we observe no benefit from increasing the coverage from 50× to 100×; actually the size statistics slightly deteriorate for Edena and Velvet, a rather unexpected and very notable result. On the other hand, when the coverage ranges between 10× and 20×, the assembly size statistics are poorer than with 50–100× coverage. In particular, having 50× versus 20× coverage clearly increases largest and N50 contig sizes. With reads containing 5% error, only Velvet succeeds in spanning over 1% of the reference (the assembly covers 66% of reference), and only with 100× read coverage. In this case, increased sequencing error makes increased coverage necessary.

### Benchmark IV Results: Effect of paired end data and error on assembly

**Figure 4:**
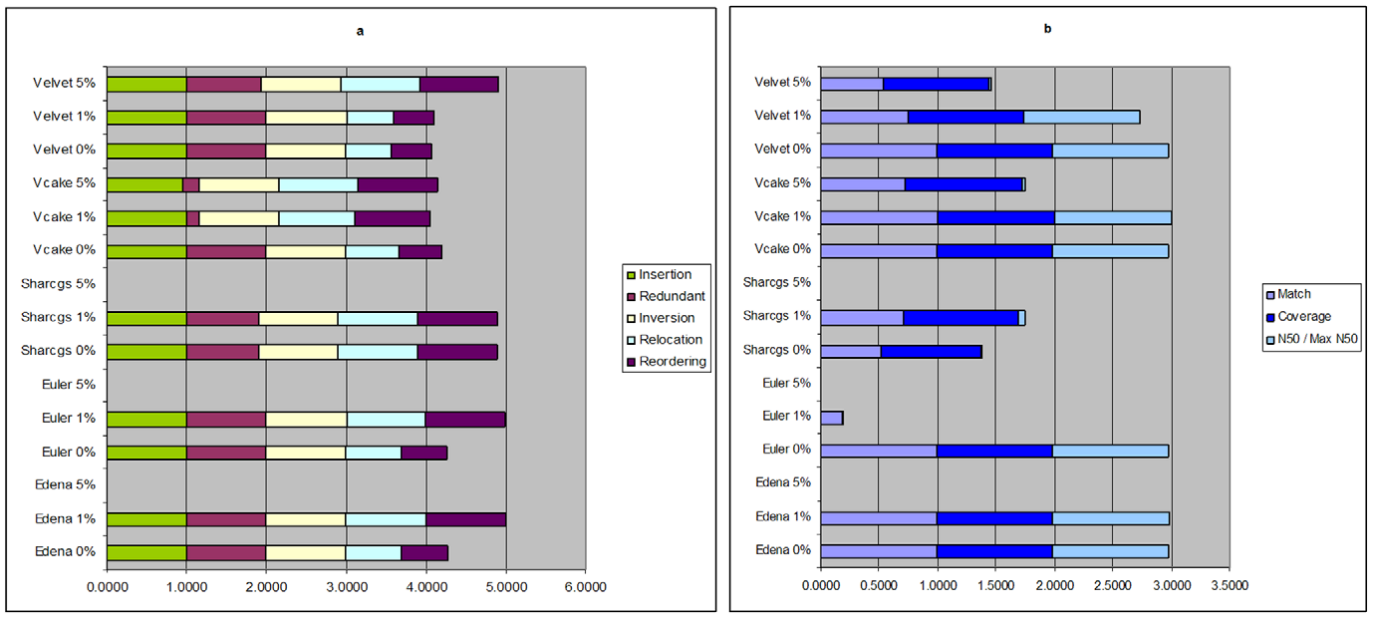
Correctness and size statistics for *O. sativa* assemblies with varying pair distances. Assembly statistics for a) correctness and b) size are shown for unpaired and paired *O. sativa* reads with distances {400, 1000, 3000} and 50× coverage, assembled by Velvet and ABySS.

*O. sativa* assembly results generated by ABySS, and Velvet for 50× coverage with pair distances (fragment sizes) of 400, 1000, and 3000 nts are shown in Fig. 4 and Table S9. With pairs it is possible to increase the coverage from 80% to nearly 100%. The number of contigs decreases and the largest and N50 contig sizes generally increase with increasing insert size, while correctness scores remain similar. In Fig. 4 we see that Velvet with 3 kb pairs and no read errors yields the best size statistics and also high correctness scores.

To study more moderate read coverages, we added 10× and 5× paired 3 kb insert size reads to 10×, 20×, and 30× coverage of linear reads. We also used 5× coverage of 0.5 kb, 1 kb, and 5 kb insert sizes coupled with 20× linear read coverage. Error rate of the reads was fixed at 1%. The results are shown in Table 1 and Table S10. In all cases, adding paired reads increased genome coverage and N50 contig size. Insert size made little difference to these results. The highest genome coverage, 86%, was reached with 30× linear combined with 10× paired 3 kb reads.

**Table 1:** *O. sativa* assembly statistics with various read coverages.

Coverage	Paired coverage	Contigs	Max	Mean	N50	ContigS	Span	Coverage	Match	Insertion	Redundant	Inversion	Relocation	Reordering
10×	10×3 kb	1,255	6,077	1,753	1,117	2,200,624	2,163,462	0.5409	0.4999	0.9840	0.9991	0.9992	0.9962	0.9998
10×	5×3 kb	257	5,172	1,291	0	331,762	331,727	0.0829	0.4967	0.9999	1.0000	0.9986	0.9953	0.9970
10×	-	1	1,038	1,038	0	1,038	1,038	0.0003	0.1106	1.0000	1.0000	1.0000	0.9990	1.0000
20×	10×3 kb	657	36,978	5,400	6,713	3,547,741	3,375,464	0.8439	0.5009	0.9525	0.9990	0.9995	0.9957	0.9981
20×	5×500 bp	1,155	14,272	2,774	2,688	3,203,871	3,154,092	0.7885	0.5004	0.9851	0.9994	0.9994	0.9980	0.9995
20×	5×1 kb	1,094	14,311	2,901	2,758	3,174,051	3,150,605	0.7877	0.5004	0.9929	0.9997	1.0000	0.9975	0.9990
20×	5×3 kb	1,084	16,728	2,894	2,723	3,137,460	3,077,230	0.7693	0.5003	0.9811	0.9997	1.0000	0.9976	0.9999
20×	5×5 kb	1,114	14,349	2,771	2,528	3,086,753	3,040,727	0.7602	0.5003	0.9853	0.9998	0.9999	0.9978	1.0000
20×	-	1,190	6,184	1,638	0	1,949,782	1,943,498	0.4859	0.4998	0.9999	0.9969	1.0000	0.9992	0.9999
30×	10×3 kb	457	57,900	7,867	12,229	3,595,118	3,437,610	0.8594	0.5014	0.9572	0.9990	0.9999	0.9949	0.9985
30×	5×3 kb	577	56,742	5,925	8,491	3,418,631	3,269,152	0.8173	0.5009	0.9563	0.9999	1.0000	0.9968	0.9993
30×	-	983	24,354	3,160	2,936	3,106,475	3,033,240	0.7583	0.5006	0.9784	0.9980	1.0000	0.9997	1.0000
**Best values**			**57,900**	**7,867**	**12,229**	**3,595,118**	**3,437,610**	**0.8594**	**0.5014**	**1.0000**	**1.0000**	**1.0000**	**0.9997**	**1.0000**

All our size and correctness statistics are shown for Velvet assemblies of reads with 1% injected errors. The read coverages vary from unpaired to combinations of unpaired and paired reads.

We studied the robustness of the results by replicating each data generation and assembly process 10 times for coverage combinations {10×, 20×, 30×} linear with {5×, 10×} paired 3 kb insert size reads. The results are shown in Table S11. The statistics are remarkably stable across different replications of the same experiment, notably the genome coverage is at most 2% different across replicates. This supports our method of presenting results based on a single run on a particular test case. The results also show Velvet produces robust assembly results at 1% error rate and uniform read sampling from the source sequence.

## Discussion

With increasing efforts to assemble genome sequences *de novo* by utilizing high-throughput sequencing technologies, there is a great interest in generating tools and strategies for the assembly task. Many assembly tools have been devised and found to be highly useful in the context of specific assembly tasks. However, choosing the best tool to use with a given sequencing and assembly strategy for a novel organism has received less attention. In the above, we presented a protocol for evaluating the chosen assembly strategy on a related model organism and applied it to several publicly available algorithms. By varying sequencing coverage, error rates and sequence composition of the target genome in a controlled setting, we estimated the extent and nature of errors that one ought to expect in a real-world setting. In addition, by pinpointing when reasonable assemblies are no longer achieved, we were able to establish limits on the read coverage, read lengths, and sequencing errors that a given assembler can tolerate.

Generally speaking, short bacterial genomes and otherwise simple sequences can be assembled accurately with many of the available assembly tools, in the presence of few sequencing errors and a high coverage of the target genomic sequence. When focusing on genomes that are architecturally more complex, such as those containing repeats or other internal structures, the assembly process becomes a less straight-forward proposition, even in the case of short genomes such as the HIV1. Additionally, in the presence of sequencing errors affecting as few as 1% of the read positions, the assembly statistics can deteriorate notably.

The evaluated tools can leave up to 20% of the reference sequence uncovered (or ∼800 k nt for *O. sativa*) when working with reads of 50 nts at 50× coverage. It is important to stress that the assembly quality and performance issues that we observed manifest themselves even when working with short genomes. In view of all these observations, it is apparent that attempting to assemble large and complex genomes (e.g. the genome of *T. cacao* with an estimated size 400 Mb) is a substantially more challenging proposition.

## Materials and Methods

### Source of genomic sequences

In order to generate representative results, we simulated input reads from viral (full length HIV1 and HHV5), bacterial (full length *E. coli*), animal (a BAC sequence from *D. melanogaster* and the complete genome sequence of the multichromosome organism *S. cerevisiae*), and plant (a 4 Mb sequence from chromosome 12 from *O. sativa*, rice) genomic reference sequences. The source genomic sequences were chosen so that they capture a range of lengths and sequence complexity. Notably, among the source genomic sequences used to generate our inputs, *O. sativa* is most similar in composition to *T. cacao*, the latter being the genome of our primary interest. Details on the reference sequences are presented in Methods S1.

### Computing environment and assembly parameters

Assemblers were run under Aix (ABySS, Sharcgs, Vcake, Velvet) and Windows (Euler, Edena). The purpose of our benchmark did not include testing the absolute or relative speed or memory requirements of the assemblers, but rather testing the correctness of the resulting assemblies. The details of the computing environments are as follows: AIX Operating System, 91 GB RAM, 4.7 GHz CPU speed; Linux Operating System, 12 GB RAM, 1.4 GHz CPU speed; Windows Operating System, 3 GB RAM, 2.4 GHz CPU speed.

In our computing environment, Euler, Sharcgs and Vcake failed to complete the assembly task for sequences larger than the HHV5 genome. All tools were run with default parameters. When *k*-mer length needed to be defined, it was set to *k* = 21 for 30 nt reads and *k* = 31 for all other reads; alternatively overlap parameter was set to *k*−1. Insert size and expected k-mer coverage for paired reads were given as parameters to Velvet.

### Evaluation protocol

We computed various size and quality statistics for each assembly. We included only contigs that were at least 1,000 nts long in our evaluation (except for HIV1, where we included all contigs at least 100 nts long). To compare the generated contigs to the reference sequence, we performed pair-wise Blast [30] searches (Blastn version 2.2.2) to map the contigs to the reference genome. We identified all contiguous matches in the reference genome for each contig (having at least 95% identity with matching length at least 100).

By using the longest match of a contig as an anchor on the reference genome, we define a *match window* for each contig: a contiguous segment of the reference sequence that is the same length as the contig, where ideally all matches for the contig are contained. Computing the first and last coordinates of the window is explained in Methods S1.

Each contig was evaluated as follows:

1. Obtain the longest match of the contig and use it as an anchor to define a match window on the reference
2. For each contig position *i*, compute its projected position in the match window, M_i_, and record its best match in the reference, i.e., the match closest to M_i_
3. Compute number of positions corresponding to relocation, insertion, and inversion mistakes
4. Compute reordering score

The values of relocation, insertion and inversion scores for the assembly are computed as the sum of positions indicated in step 3 above, divided by the total assembly size. The reordering score for the assembly is defined as a weighted average of each contig's reordering scores, where weight equals contig length. Coverage, match score, and redundancy score are computed after recording every contig's best matching positions in the reference. The correctness scores and size statistics are described below, further details are available upon request.

### Correctness scores

Insertion score for a contig is one minus the fraction of contig positions that do not match the reference. Relocation score for a contig is one minus the fraction of contig positions that match the reference outside the match window. Inversion score for a contig is the fraction of contig positions with matches sharing their orientation with the contig's, see Methods S1. Reordering score is computed as one minus the fraction of contig position pairs that are in conflicting order in the contig and the reference (to ensure computational efficiency, every 100 positions from the contigs were sampled to perform this evaluation, except for HIV1 every 10 positions due to its shorter contigs). Redundancy score is one minus the fraction of assembly positions that match to a reference position already covered by one or more other assembly positions.

### Size statistics

The N50 contig size is the largest contig length such that at least 50% *of the total length of the reference sequence* can be obtained by considering contigs of at least this length. N50 is a measure of the fragmentation of the assembly. Span denotes the length of the reference sequence that has contig positions from the assembly aligned to it, whether the alignments are correct or not. Coverage refers to the percentage: span divided by reference sequence length. Match score rewards for matches and penalizes for gaps, taking their lengths into account, and is defined as ½{ Σ_s_(|u_s_|/n)^2^+(1−Σ_t_(|v_t_|/n)^2^ ) }, where the reference length n is divided into alternating non-overlapping match segments (u) and gap segments (v), n = Σ_s_u_s_+Σ_t_v_t_. Gap segments consist of adjacent positions in the reference sequence with no contigs positions aligned to them.

### Additional methods

Assembly evaluation details, reference sequences, simulated data sets and a tool for generating simulated reads from a given sequence are available in Methods S1. Tables S1, S2, S3, S4, S5, S6, S7, S8, S9, S10, and S11 contain assembly statistics for each studied genome sequence.

## Supporting Information

Methods S1 **Details on the applied assembly and evaluation protocols, and lists the assembly programs and reference sequences used.**
(DOC)

Table S1 **HIV1 assembly details.**
(XLS)

Table S2 **BAC assembly details.**
(XLS)

Table S3 **HHV5 assembly details.**
(XLS)

Table S4 ***O. sativa*** **assembly details.**
(XLS)

Table S5 ***E. coli*** **assembly details.**
(XLS)

Table S6 ***S. cerevisiae*** **assembly details.**
(XLS)

Table S7 ***O. sativa*** **assembly details with varying read lengths.**
(XLS)

Table S8 ***O. sativa*** **assembly details with varying linear read coverages.**
(XLS)

Table S9 ***O. sativa*** **assembly details with varying pair distances.**
(XLS)

Table S10 ***O. sativa*** **assembly details with varying read coverages and pair distances.**
(XLS)

Table S11 **Robustness results for** ***O. sativa*** **assemblies with varying read coverage combinations.**
(XLS)
