# Correction: Evaluation of Methods for *De Novo* Genome Assembly from High-Throughput Sequencing Reads Reveals Dependencies That Affect the Quality of the Results

**DOI:** 10.1371/annotation/bb125f93-80d3-4dd1-adfe-03d9fb740f3b

**Published:** 2011-10-05

**Authors:** Niina Haiminen, David N. Kuhn, Laxmi Parida, Isidore Rigoutsos

The funding information should read as follows: IBM funded this study through the employment, at the time of the study, of Isidore Rigoutsos, Niina Haiminen, and Laxmi Parida. Between them they conceived, designed and performed the experiments, analyzed the data and designed the assembly evaluation protocol. David Kuhn is employed by USDA-ARS SHRS and received no funding for this study from IBM. He participated in the analysis and interpretation of data, and the writing of the paper.

