# Correction: Evaluation of Methods for *De Novo* Genome Assembly from High-Throughput Sequencing Reads Reveals Dependencies That Affect the Quality of the Results

**DOI:** 10.1371/annotation/176d83be-ed67-4205-9265-7208792d3dcf

**Published:** 2011-10-05

**Authors:** Niina Haiminen, David N. Kuhn, Laxmi Parida, Isidore Rigoutsos

The Competing Interests statement should read as follows: The authors have read the journal's policy on competing interests and have the following conflicts. Niina Haiminen and Laxmi Parida are employees of IBM. Isidore Rigoutsos was employed by IBM at the time of the study. There are no patents, products in development or marketed products to declare. This does not alter the authors' adherence to all the PLoS ONE policies on sharing data and materials, as detailed online in the guide for authors.

