# Supplementary material for: Evaluation of Methods for De Novo Genome Assembly from High-Throughput Sequencing Reads Reveals Dependencies That Affect the Quality of the Results
Source: PLoS One. 2011 Sep 7;6(9):e24182. doi: 10.1371/journal.pone.0024182 (PMC3168497; doi:10.1371/journal.pone.0024182)
Supplement: Methods S1 — Details on the applied assembly and evaluation protocols, and lists the assembly programs and reference sequences used. (DOC) [file pone.0024182.s001.doc]

**Supporting Methods for**

**“Evaluation of Methods for de novo Genome Assembly from High-throughput Sequencing Reads Reveals Dependencies that Affect the Quality of the Results”**

This document contains details on the applied assembly and evaluation protocols, and lists the assembly programs and reference sequences used.

**METHOD DETAILS**

**Read generation**

For each given sequence, we generated reads of a fixed length with uniformly random distribution of locations across the sequence. The reads were then sorted by sequence (so as not to give them in the correct order to the assembly algorithms), and each read given a fasta-format label corresponding to its rank. Paired reads were generated from opposite ends of a sequence fragment of given length *L*, the insert size chosen uniformly at random from ]*L*-50, *L*+50[. No per-base quality information was utilized in this study.

**Assembly**

The assembly algorithms were run with default parameters. The parameters are discussed below. Each assembler was given the fasta-format reads as input, and when *k*-mer length needed to be provided to the algorithm, it was set to *k*=31 for 50 nt reads, and to *k*=21 for 30 nt reads, corresponding to 2/3 of the read length. The *k*-mer lengths we used gave reasonable results. However, varying *k*-mer length may have significant impact on the results. In this study we kept this parameter fixed and instead focused on and compared the following attributes: genome composition, sequencing error rates, read lengths, coverages, and paired insert sizes. Velvet required defining insert size *L* and average *k*-mer coverage when assembling paired reads: these were given as parameters. Abyss required the minimum number of pairs *n* as input, example value *n*=10 was used.

For paired reads, Velvet gave as output scaffolds containing contigs joined with stretches of N’s. These scaffolds were broken into contigs at all locations where N’s were found, and the contigs were evaluated independently of each other. Only contigs at least 1,000 nt long were considered in the evaluation.

**Assembly evaluation**

For each assembly, we aligned the set of contigs to the reference genome by using the following Blast command (version 2.2.2), for example for *E. coli* assemblies:

**blastall -p blastn -d ecoli -m 8 -F F -n T -i contigs.fasta -o results.txt**

Here “ecoli” refers to a Blast database built from the reference *E. coli* sequence by running the Blast command **formatdb**. All matches for each contig was extracted. If a match had at least 95% identity with the reference and length at least 100, it was processed further.

**Defining match window coordinates**

Match window coordinates are computed for each contig, based on the coordinates of the longest match for the contig. Match window corresponds to a segment of the reference sequence, where the entire contig will map if correctly assembled, thus its length equals the contig’s length.

The coordinates of a match window are determined by the longest match of the contig against the reference, and are such that if the longest match is placed correctly on the reference, all other correct matches will fit in the window. Let us have a match spanning positions [x,y] in the reference and positions [a,b] in a contig of length L, both in the same orientation. The positions of the match window will be [S, E] = [ x – a – 1, y + L – b ], and the contig’s orientation ‘+’. If the contig and reference have opposite orientations in the match, we have [S, E] = [ x – (L – b), y + a – 1 ], and the contig’s orientation is ‘-‘.

The projected position of a contig position *i* is M_i_ =S+*i*-1 when the contig has orientation ‘+’, and M_i_ =E-*i*+1 when the contig has orientation ‘-‘. It determines where the contig position should fit in the reference, given that the longest match for the contig was correct.

**ASSEMBLY PROGRAMS USED**

| **Program** | **Version** | **Location** |
| --- | --- | --- |
| ABySS | 1.1.1 | http://www.bcgsc.ca/platform/bioinfo/software/abyss |
| Edena | 2.1.1 | http://www.genomic.ch/edena.php |
| Euler-SR | 1.1.2 | http://euler-assembler.ucsd.edu/portal/ |
| Sharcgs | - | http://sharcgs.molgen.mpg.de/index.shtml |
| Vcake | 1.0 | http://sourceforge.net/projects/vcake/ |
| Velvet | 0.7.28 | http://www.ebi.ac.uk/~zerbino/velvet/ |

**REFERENCE SEQUENCES USED**

| **Organism** | **Region** | **Size** | **Accession / Location** |
| --- | --- | --- | --- |
| HIV 1 | Genome | 9.2 kb | NC_001802 |
| *D. melanogaster* | BACs from chr. 2L | 19.7 kb | http://sharcgs.molgen.mpg.de/data/AC006575.seq |
| HHV 5 | Genome | 230.3 kb | NC_001347 |
| *E. coli* | Genome | 4.6 Mb | NC_000913 |
| *O. sativa* | Genome segment | 4.0 Mb | NC_008405 chromosome 12; 1—4,000,000 bp |
| *S. cerevisiae* | Genome | 12.1 Mb | NC_001133—NC_001148 |
